# Managing Hostile Aortic Anatomies Using an Extended-Length Introducer Sheath During Transfemoral Transcatheter Aortic Valve Replacement: Rationale and Clinical Outcomes

**DOI:** 10.1016/j.shj.2025.100737

**Published:** 2025-10-08

**Authors:** Yusuke Kobari, Davorka Lulic, Arif A. Khokhar, Yinghao Lim, Tau Sarra Hartikainen, Laurence Campens, Yannick Willemen, Gintautas Bieliauskas, Ole De Backer

**Affiliations:** a Department of Cardiology, The Heart Center, Rigshospitalet, Copenhagen, Denmark; b Department of Clinical Medicine, University of Copenhagen, Copenhagen, Denmark

**Keywords:** Aortic stenosis, Extended length introducer sheath, Stroke, Transcatheter aortic valve replacement, Vascular complication

## Abstract

**Background:**

Transfemoral (TF) access is the safest and guideline-recommended approach for transcatheter aortic valve replacement (TAVR). However, in patients with hostile aortic anatomies, the procedure may carry increased risks of vascular or cerebrovascular complications. The objective of this article is, therefore, to evaluate the safety and efficacy of using the extended-length DrySeal Flex introducer sheath (Gore, USA) for TF-TAVR in high-risk patients with challenging aortic anatomies.

**Methods:**

We conducted a retrospective, single-center cohort study including all consecutive patients who underwent TF-TAVR with the 65-cm DrySeal sheath between 2021 and 2025. We analyzed the indications for sheath use and the following clinical outcomes: all-cause mortality, stroke, and major vascular complications as defined by Valve Academic Research Consortium-3 criteria.

**Results:**

The 65-cm DrySeal sheath was used in 200 patients (median age 80 years; median Society of Thoracic Surgeons score 5.4%) out of 2430 (8.2%) TF-TAVR procedures performed. This approach was selected to address challenges posed by aortic arch calcification (28%), heavily atheromatous or shaggy aorta (20%), acute aortic angulation (26%), tortuosity (36%), and aortic coarctation (8%). In this high-risk cohort, all-cause mortality was 0.5%, stroke occurred in 1.5% (including 0.5% with disabling stroke), and major vascular complications were observed in 1.0%. These outcomes were comparable to those seen in lower-risk patients undergoing TF-TAVR using conventional approaches.

**Conclusions:**

In patients with hostile aortic anatomies, the extended-length DrySeal Flex introducer sheath facilitated safe and effective TAV delivery and was associated with a low rate of periprocedural complications, including cerebrovascular events. Its use may help mitigate the risks traditionally associated with hostile aortic anatomies.

## Highlights

• The extended-length DrySeal sheath enabled safe and effective TAV delivery in hostile anatomies.
• This strategy was associated with a very low rate of periprocedural complications, including cerebrovascular events.
• Its use may mitigate the risks traditionally linked to hostile aortic anatomies during transfemoral TAVR.

## Introduction

Transcatheter aortic valve replacement (TAVR) via the transfemoral (TF) approach is the preferred and guideline-recommended treatment for older patients with severe aortic stenosis (AS).^1^^,^^2^ Over recent years, technological advancements and procedural improvements have broadened the eligibility for TF-TAVR, which consistently demonstrates favorable clinical outcomes compared to alternative access routes.^3^^,^^4^ However, a subset of patients presents with hostile aortic anatomies—characterized by significant tortuosity, severe calcifications, and extensive atheromatous (shaggy) disease—which increases the risk of mechanical aortic injury and cerebrovascular events (CVEs).^5^, ^6^, ^7^, ^8^ The proximal descending aorta, aortic arch, and ascending aortic root are particularly susceptible due to the need for repeated manipulations with wires, catheters, and the TAV delivery system.

Efforts to mitigate these risks have included various procedural strategies and device developments, but with limited success. Recent randomized controlled trials assessing cerebral embolic protection devices (CEPDs) were unable to demonstrate their efficacy in reducing CVE, underscoring the need for alternative or adjunctive approaches.^9^^,^^10^ One emerging strategy involves the use of an extended-length introducer sheath, which bypasses the vulnerable segments of the proximal aorta while maintaining a femoral access route. However, data on this approach remain limited.^11^

This study aimed to evaluate the safety and efficacy of using an extended-length introducer sheath in a large consecutive cohort of patients with hostile aortic anatomies undergoing TF-TAVR.

## Methods

### Study Population

Between 2021 and 2025, 2430 patients underwent TF-TAVR at our center. Of these, 200 patients with hostile aortic anatomies underwent TF-TAVR with self-expanding valves using a 65 cm-long DrySeal Flex introducer sheath (GORE Medical, USA) and were included in this analysis. Self-expanding valves were used in 90% of all TAVR. Patients who underwent TAVR via alternative access routes were excluded. The study was conducted in accordance with the Declaration of Helsinki, with ethical approval obtained from the local institutional review board.

### DrySeal Flex Introducer Sheath

The GORE DrySeal Flex introducer sheath is a fixed-diameter introducer sheath originally developed for aortic endovascular procedures. It features a hydrophilic coating, a long-tapered dilator, and enhanced flexibility combined with kink resistance, making it well-suited for navigating challenging vascular anatomies. Its unique hemostatic valve mechanism prevents blood loss during device insertions and removals and allows for simultaneous multiple catheter insertions, if needed. Available in various diameters (14-26F, inner diameter) and lengths (33 and 65 cm), the sheath is compatible with all commercially available TAV platforms.

### Indications for Use of an Extended-Length Introducer Sheath During TF-TAVR

**Figure 1:**
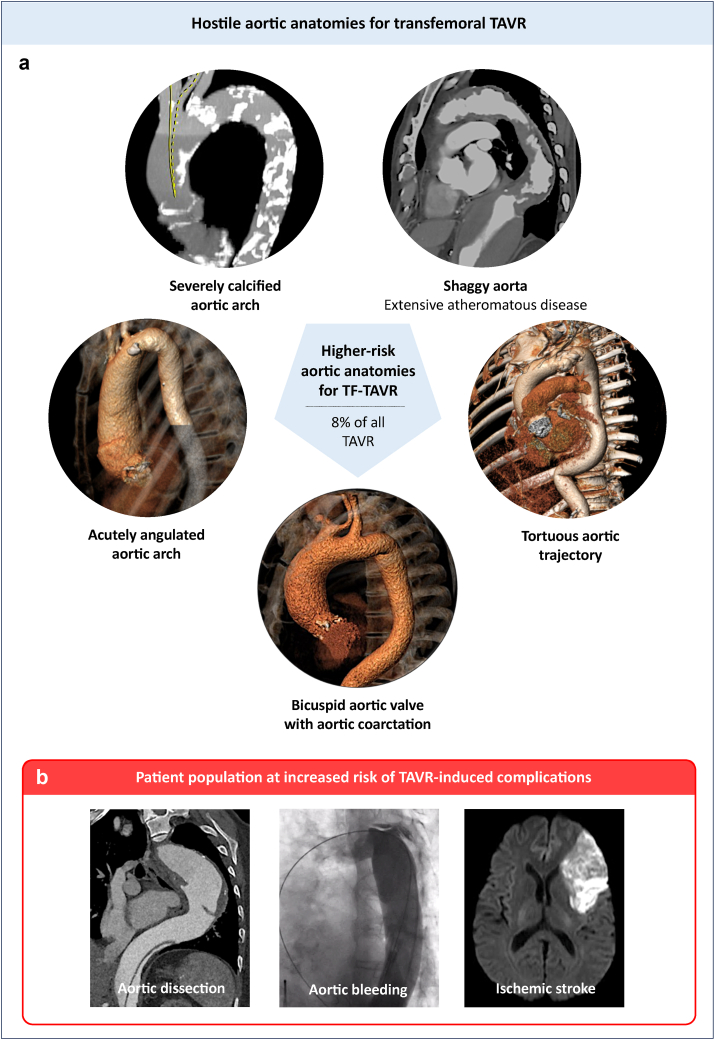
**Hostile aortic anatomies for transfemoral TAVR (a) and possible complications (b)**. **(a)** Higher-risk aortic anatomies for TF-TAVR, especially in case of use of nonsteerable self-expanding valves. One or more high-risk features indicating a hostile aortic complex was present in 8% of all TAVR patients. **(b)** Aortic injury/dissection, aortic bleeding and ischemic stroke are the main complications to avoid. Abbreviations: TAVR, transcatheter aortic valve replacement; TF, transfemoral.

In patients scheduled for TF-TAVR, preprocedural computed tomography analysis of the iliofemoral access sites and the aortic complex (descending aorta, arch, and ascending aorta) is essential. The extent of tortuosity, calcification, atheromatous burden, presence of thrombi, aneurysms, or prior grafts/stents are carefully evaluated. The decision to employ the 65 cm DrySeal sheath was based on the presence of one or more high-risk features indicating a hostile aortic complex: severely calcified aortic arch, extensive atheromatous disease (shaggy aorta), acutely angulated aortic arch, significant aortic tortuosity or kinking, congenital or acquired abnormalities of the aortic anatomy (Figure 1). The decision to use the extended-length DrySeal sheath was made during the Heart Team meeting based on preprocedural computed tomography imaging. Even when alternative, non-TF approaches were technically feasible, we consistently prioritized TF-TAVR in line with current guideline recommendations, as our center follows a standardized workflow designed to allow patients to undergo TF-TAVR with local anesthesia, ambulate within 3 hours after TAVR, and be discharged the following day.

### Procedural Steps

Access was obtained using contemporary ultrasound-guided techniques. After inserting a 6F sheath, a preclosure suture device (ProStyle, Abbott, USA) was used, followed by an 8F sheath. Closure strategies adhered to the TAVR MultiCLOSE algorithm, which combines suture-based and plug-based closure devices. In cases requiring extensive iliofemoral disease treatment, percutaneous angioplasty and/or intravascular lithotripsy were performed prior to insertion of the introducer sheath.

**Figure 2:**
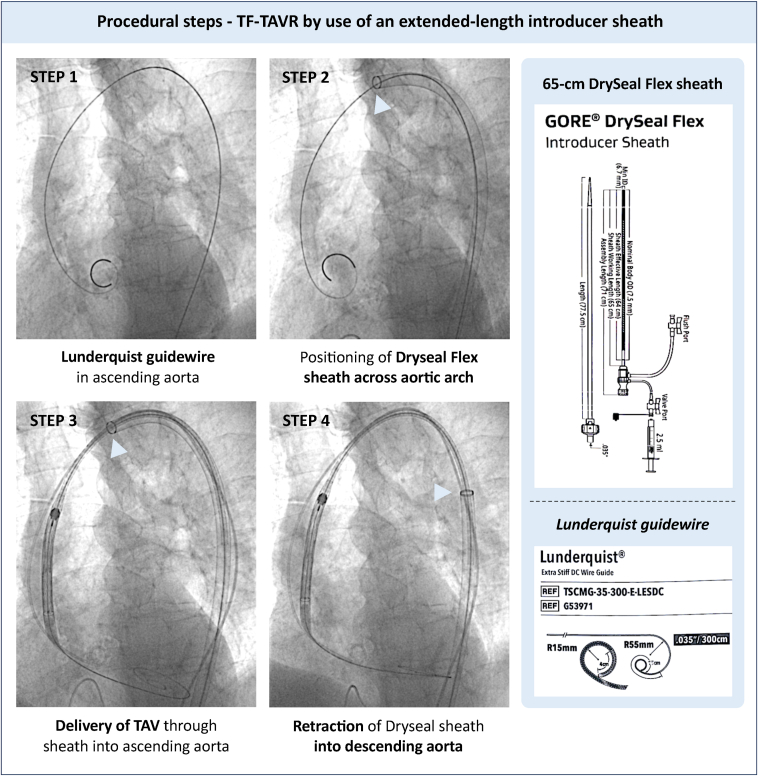
**Procedural steps - TF-TAVR by use of an extended-length introducer sheath**. A standardized technique for use of a 65 cm-long DrySeal Flex introducer sheath for cases with hostile aortic anatomy was developed and adopted. A detailed step-by-step description can be found in the Methods section. Abbreviations: TAV, transcatheter aortic valve; TAVR, transcatheter aortic valve replacement; TF, transfemoral.

A standardized technique was developed and adopted for the use of a 65-cm-long DrySeal Flex introducer sheath for cases with hostile aortic anatomy, which is described in Figure 2.

#### Step 1

Advance a conventional 0.035” guidewire into the ascending aortic root. In challenging anatomies, especially with extreme tortuosity, the use of a 0.035” Advantage GlideWire (Terumo, Japan) is recommended for safer, controlled navigation with adequate support. Exchange the 0.035” guidewire for a stiff Lunderquist double-curved wire (Cook Medical, USA), positioned just above the aortic valve within the ascending aorta.

#### Step 2

Insert the extended-length DrySeal sheath over the Lunderquist wire, advancing it across the arch into the ascending aorta. For larger sheath diameters, predilation of the femoral access site may be considered. In shorter patients, caution is necessary to prevent the sheath dilator from engaging or crossing the aortic valve.

#### Step 3

Deliver the TAV system through the DrySeal sheath into the ascending aorta, herewith avoiding contact with the hostile aortic anatomy.

#### Step 4

After crossing the aortic valve with the TAV, the sheath can be retracted toward the top of the descending aorta if the additional support/stiffness is thought to interfere with catheter coaxiality during valve deployment. Following valve implantation, carefully retract the TAV delivery system. For self-expandable TAVs, manual recapture in the ascending aorta is recommended before withdrawing the TAV delivery system into the DrySeal sheath.

### Study Outcomes

The main study endpoints were prespecified and included all-cause mortality, stroke (disabling and nondisabling), and major vascular complication (access-site related and aortic injury) which occurred within 72 hours periprocedurally. These outcomes were assessed according to the Valve Academic Research Consortium-3 criteria^12^ and compared with those obtained in patients undergoing TF-TAVR without the use of an extended-length sheath during the same period at our institution.

### Statistical Analysis

Categorical variables are presented as numbers and percentages, while continuous variables are expressed as medians with interquartile ranges. Differences in clinical outcomes were compared using two-sided chi-square tests. A *p* value less than 0.05 was considered statistically significant. All analyses were conducted using SPSS version 29.0 (IBM, New York, USA).

## Results

### Baseline Clinical and Procedural Characteristics

Among 2430 patients who underwent TF-TAVR between 2021 and 2025 at our center, the extended-length 65 cm-long DrySeal Flex introducer sheath was used in 200 consecutive patients (8.2%) with a hostile aortic anatomy. Compared with the conventional approach, patients treated with the long DrySeal sheath had a higher prevalence of peripheral arterial disease (20.5 vs. 8.7%; *p* < 0.001) and prior stroke (15.5 vs. 9.6%; *p* = 0.008), more frequently presented with bicuspid aortic valve (30.0 vs. 11.0%; <0.001), and consequently exhibited a higher median Society of Thoracic Surgeons score (5.4 vs. 4.0%; *p* < 0.001), reflecting the high-risk nature of this patient cohort (Table 1).

**Table 1:** Patient population

	Long DrySeal approach	Conventional approach	*p* value
	N = 200	N = 2230	
Clinical variables			
Age, y	80 (76-84)	79 (76-83)	0.074
Female	72 (36.0%)	827 (37.8%)	0.622
Arterial hypertension	143 (71.5%)	1676 (76.5%)	0.110
Diabetes mellitus	30 (15.0%)	518 (23.7%)	0.005
Body mass index, kg/m^2^	25.0 (22.9-28.4)	26.2 (23.5-30.0)	0.004
Coronary artery disease	60 (30.0%)	784 (35.8%)	0.100
Peripheral arterial disease	41 (20.5%)	191 (8.7%)	<0.001
Impaired renal function∗	75 (37.5%)	791 (36.1%)	0.697
Atrial fibrillation	72 (36.0%)	769 (35.1%)	0.802
Prior stroke	31 (15.5%)	211 (9.6%)	0.008
Bicuspid aortic valve	60 (30.0%)	241 (11.0%)	<0.001
Prior surgical aortic valve replacement	13 (6.5%)	102 (4.7%)	0.244
STS surgical risk score, %	5.4 (3.5-6.8)	4.0 (3.2-4.9)	<0.001
Procedural variables			
TAV type			<0.001
Evolut R/PRO/FX	127 (63.5%)	736 (33.6%)	
Portico/Navitor series	54 (27.0%)	847 (38.7%)	
ACURATE neo2/Prime	6 (3.0%)	349 (15.9%)	
Others	13 (6.5%)	258 (11.8%)	
Predilatation	181 (90.5%)	1942 (88.7%)	0.433
Postdilatation	116 (58.0%)	935 (42.7%)	<0.001
In-line approach	0 (0.0%)	538 (32.0%)	<0.001
External sheath approach	200 (100.0%)	1142 (68.0%)	
DrySeal introducer sheath size			
18F	81 (40.5%)		
20F	90 (45.0%)		
22F	28 (14.0%)		
24F	1 (0.5%)		

Abbreviations: STS, Society of Thoracic Surgeons; TAV, transcatheter aortic valve.

∗ Impaired renal function defined as estimated glomerular filtration rate <30 mL/min/1.73 m^2^.

### Indications for the Use of an Extended-Length DrySeal Introducer Sheath

**Figure 3:**
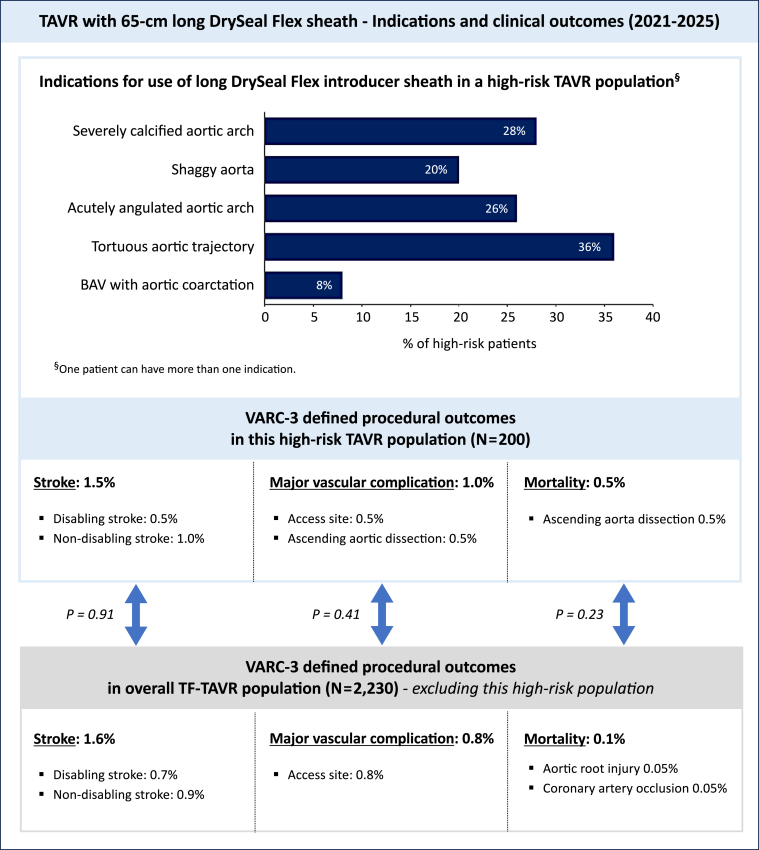
**Indications and clinical outcomes for TF-TAVR using a 65-cm long DrySeal Flex sheath**. Indications and VARC-3-defined clinical outcomes in a patient cohort of 200 patients with a hostile aortic anatomy treated by TF-TAVR using an extended-length DrySeal Flex introducer sheath. These outcomes were compared to TF-TAVR cases using a conventional approach. Abbreviations: BAV, bicuspid aortic valve; TAVR, transcatheter aortic valve replacement; TF, transfemoral; VARC, Valve Academic Research Consortium.

The 65 cm-long DrySeal sheath enabled TF delivery and implantation of the TAV in 100% of patients. The primary indications for selecting the extended-length DrySeal included challenging aortic anatomies: severe aortic arch calcification (28%), heavily atheromatous or shaggy aorta (20%), acute aortic angulation (26%), tortuosity (36%), and bicuspid AS with aortic coarctation (8%) (Figures 1 and 3).

The most frequently used sheath size was 18F (45%), followed by 20F (41%) and 22F (14%). The Evolut valve (Medtronic, USA) was the most commonly implanted TAV, used in 63.5% of cases. Predilatation was performed routinely (91%), and postdilatation was performed in 58% of cases, due to the high prevalence of bicuspid AS and valve-in-valve TAVR.

### Vascular Closure

The most commonly used vascular closure technique combined a single ProStyle device with an AngioSeal (Terumo, Japan), achieving successful closure in 178 of 200 patients (89.3%). In 18 patients (9.0%), a second ProStyle was used before AngioSeal deployment, and bailout vascular closure with a MANTA device was employed in 4 patients (2.0%). A major access-site-related vascular complication was noted in 1 out of 200 patients (0.5%).

### Valve Academic Research Consortium-3 Defined Procedural Outcomes

Despite the high-risk nature of the patient cohort with hostile aortic anatomy, the incidences of all stroke (1.5%), disabling stroke (0.5%), and major vascular complications (1.0%) remained low. There was a single case with procedural mortality (0.5%), attributed to an ascending aortic dissection caused by multiple Evolut recaptures and repositionings in a patient with a severely calcified bicuspid AS and a horizontal aorta with aortopathy. When comparing these outcomes with patients who underwent TF-TAVR using conventional techniques without the extended-length sheath, there was no statistically significant difference in stroke rates (1.5 vs. 1.6%; *p* = 0.91), major vascular complications (1.0 vs. 0.8%; *p* = 0.41), or all-cause mortality (0.5% vs. 0.1; *p* = 0.23) (Figure 3).

## Discussion

In this study, we evaluated the safety and efficacy of using the extended-length 65-cm DrySeal Flex introducer sheath for TF-TAVR in 200 high-risk patients with challenging aortic anatomies. Our key findings were: (1) TF-TAVR was feasible in all cases despite hostile anatomies; (2) the most common reasons for selecting an extended-length DrySeal sheath included severe aortic tortuosity (36%), a severely calcified arch (28%), an acutely angulated arch (26%), and extensive atheroma or “shaggy” aorta (20%); (3) periprocedural complication rates were low, with stroke and major vascular complications occurring in 1.5% and 1.0% of patients, respectively; and (4) these complication rates were comparable to those seen in lower-risk patients undergoing conventional TF-TAVR.

The global increase in TAVR volume has driven continuous advancements in TAVR technology. Contemporary TAVR devices feature smaller insertion profiles, integrated sheaths and/or expandable sheath options as well as improved materials for vessel entry and enhanced flexibility and support during valve delivery.^13^^,^^14^ These innovations, along with refined vascular access and closure techniques, have established percutaneous TF-TAVR as the preferred and guideline-recommended approach for suitable patients.^1^^,^^2^ However, conventional introducer sheaths, typically measuring 30–40 cm in length, are designed primarily to traverse the iliofemoral arteries and extend just beyond the aortoiliac bifurcation. They do not adequately address challenges within the more proximal segments—such as the descending thoracic aorta, arch, and ascending aorta—that often pose significant obstacles in patients with complex anatomies. Conditions like severe tortuosity, kinking, heavy atheromatous or calcific burden, and prior endovascular grafts or stents can hinder device delivery and increase the risk of mechanical injury or embolic events.^5^, ^6^, ^7^, ^8^

As a consequence, a strategy using a 65-cm-long DrySeal Flex introducer sheath for TF-TAVR in patients with a hostile aortic anatomy was adopted at our center since 2021. A similar approach has been employed in transcatheter pulmonary valve implantation, where an extended-length DrySeal sheath demonstrated safety and efficiency, leading to fewer complications and shorter delivery times compared to conventional methods.^15^^,^^16^ Beyond these indications, prior reports have also described its use in facilitating leaflet modification procedures with multiple catheters through a single access, as well as in transcaval TAVR, further highlighting the versatility of the system. In the realm of TAVR, however, literature on the use of extended-length sheaths remains limited. Notably, the latest-generation Trilogy TAV (JenaValve Technologies, California, USA) utilizes an 85-cm-long sheath to facilitate valve delivery, further underscoring the potential of longer sheaths in the TAVR field.^17^

### Protection From Aortic Complications During TAVR

In patients with highly tortuous or angulated aortas—features encountered in 60% of patients in our high-risk cohort—the use of a 65-cm DrySeal sheath proved instrumental. Such anatomies often impede catheter and device navigation, requiring excessive force or multiple repositioning maneuvers, which increase the risk of aortic wall injury. Stiffer guidewires are frequently employed to overcome these hurdles but can heighten the potential for mechanical injury or ventricular perforation. Conversely, an extended-length sheath can straighten tortuous segments, enabling more controlled and safer catheter manipulations, potentially reducing procedural complexity and complication risk.

Furthermore, the extended-length DrySeal sheath provides advantages when traversing congenital or acquired obstructions within the aorta, such as coarctations (present in 9% of our bicuspid AS patients) and previous aortic interventions with grafts or stents. These scenarios complicate device passage and exchange, increasing the risk of injury or dislodgment. The sheath's stability and length help facilitate safe navigation across such obstacles, reducing procedural time and risk.

Importantly, we also demonstrated that using an extended-length introducer sheath allows for these aortic challenges and obstacles to be safely and successfully mitigated, without paying a penalty in the rate of vascular access site complications.

### Protection From CVE During TAVR

Periprocedural CVE during TAVR can result from multiple factors. Accumulating evidence indicates that the presence of significant aortic wall thrombus and/or protruding atheroma is associated with an increased risk of CVE.^5^^,^^7^ In a prospective study of 641 consecutive TF-TAVR patients, significant aortic wall thrombus—detected in 11.4%—was linked with higher rates of ischemic stroke (odds ratio [OR]: 5.66; 95% CI: 2.00–15.30; *p* = 0.002) and all-cause mortality (OR: 4.66; 95% CI: 1.80–11.30; *p* = 0.002).^5^ Another study involving 977 patients found that protruding aortic atheroma (≥3 mm thickness) or ulcerated atheroma markedly increased the risk of periprocedural stroke (adjusted OR: 2.55; 95% CI: 1.37–4.74), with an even higher risk when such lesions were located in the aortic arch (adjusted OR: 3.86; 95% CI: 1.69–8.83).^7^ These findings highlight the vulnerability of the aortic arch to dislodgment of friable atheromatous material during repeated passage of wires, catheters, balloons, and TAVs, which can precipitate cerebral embolic events. Although various strategies and CEPDs have been developed to mitigate these risks, large-scale randomized controlled trials have thus far failed to demonstrate clear benefit.^9^^,^^10^

In our cohort, half of the patients exhibited significant calcification or heavy atheromatous (“shaggy”) aorta, with 15% having a history of prior stroke. Despite this high-risk profile, the incidence of stroke following TF-TAVR was only 1.5%, with disabling stroke occurring in just 0.5%. Comparatively, the BHF PROTECT-TAVR (British Heart Foundation Randomised Clinical Trial of Cerebral Embolic Protection in Transcatheter Aortic Valve Implantation) trial reported a stroke rate of 2.1% and disabling strokes in 1.2%, despite the use of a dedicated CEPD. We hypothesize that the use of an extended-length sheath may provide a protective effect by physically excluding the vulnerable aortic segment from repeated catheter and device exchanges. Additionally, by overcoming significant aortic tortuosity or angulations, the extended-length sheath facilitates more controlled and precise TAV deployment, thereby minimizing the need for valve repositioning maneuvers that could increase embolic risk.

### Technical Considerations When Using an Extended-Length Introducer Sheath

Despite its advantages, certain technical factors must be considered to avoid complications. The use of a longer sheath increases the risk for air embolism or thrombus formation. Meticulous flushing and monitoring of activated clotting time are recommended to minimize these risks.

The sheath tip’s position may vary depending on patient height and vessel tortuosity; in some rare cases—typically male patients taller than 185-190 cm—the tip may not reach the ascending aorta and may terminate within the arch. When the sheath tip abuts the outer curvature of the arch, caution is needed during catheter and device exchanges to prevent aortic wall injury. In such scenarios, withdrawing the DrySeal sheath tip a little into the descending aorta is advisable.

While increased support from the extended sheath facilitates TAV delivery, its added stiffness can affect the trajectory (coaxiality) of the valve crossing, especially in anatomies with a horizontal or vertical aorta. The latest-generation self-expanding TAVs are designed to be more flexible and traverse the aortic annulus more along the outer curvature. In certain anatomies and depending on the DrySeal sheath position, the TAV may be pushed even more into the aortic outer curvature, which may impact the TAV coaxiality, implantation mechanics, and subsequent implantation depth. In these cases, once the TAV has successfully crossed the aortic valve, the extended-length introducer sheath can then be withdrawn back toward the descending aorta.

Finally, delivering a TAV through an extended-length sheath usually requires at least an 18F diameter, which can go up to 24F depending on the device type. Although this might raise concerns about vascular access site complications, our experience suggests that combining a single Prostyle preclosure device with an AngioSeal was sufficient for successful vascular closure in 90% of cases.

### Valve Selection and Use of the Extended-Length DrySeal Sheath

The extended-length DrySeal sheath is technically compatible with all commercially available TAVR platforms. However, its adoption was more frequent in self-expanding valves. This reflects 2 practical considerations. First, balloon-expandable systems generally require larger sheath sizes (≥24F) compared with self-expanding valves when using a long DrySeal sheath. The larger sheath diameter may increase the risk of vascular complications, which limits the perceived benefit of an extended-length DrySeal sheath in this setting. Second, balloon-expandable delivery systems incorporate a flex mechanism that facilitates advancement through the aortic arch and minimizes device–arch interaction, thereby reducing the need for an extended sheath. As a result, the DrySeal was most commonly used with self-expanding valves in our cohort, in which Evolut accounted for 63.5% of procedures. These characteristics of device design and sheath requirements likely influenced operator preference and should be considered when extrapolating these findings to other TAVs.

Importantly, the extended-length DrySeal sheath allows the TF approach to remain feasible, thereby avoiding the need for an alternative, non-TF approach. This is clinically relevant because TF-TAVR is consistently associated with lower complication rates, shorter hospital stays, and faster recovery compared with non-TF approaches. Moreover, it may reduce bleeding, particularly in patients with a large body habitus undergoing TAVR with self-expanding valves, as it eliminates the need for sheath exchange in the groin (in particular when performing predilatation and/or postdilatation). Thus, the extended-length sheath can be considered a practical strategy to preserve the preferred TF approach even in patients with hostile aortic anatomies.

### Study Limitations

Several limitations should be acknowledged. First, this was a retrospective, single-center observational study, which introduces potential biases and confounding variables. Second, the decision to use the 65-cm DrySeal introducer sheath was at the discretion of the operating physicians, possibly limiting the generalizability of the findings. Although we employed specific criteria to guide sheath selection, prospective studies are needed to better identify patient and anatomical factors that predict a higher risk of periprocedural complications with conventional approaches. Additionally, because current-generation TAV devices generally require at least an 18F sheath—up to 24F depending on the device—this approach may have limited utility in patients with borderline iliofemoral vessel diameters, where an expandable sheath or integrated in-line sheath may be preferred. Finally, our findings are specific to the use of the GORE DrySeal sheath and cannot be extrapolated to other long sheath systems with different materials or mechanical properties.

## Conclusions

The extended-length 65 cm DrySeal introducer sheath effectively facilitated TF-TAVR in patients with complex, hostile aortic anatomies, with notably low rates of vascular injury and CVEs in this high-risk cohort. This strategy appears to be a safe and reliable approach for overcoming anatomical challenges during TF-TAVR. Further prospective studies are necessary to confirm its safety and efficacy across broader populations.

## Ethics Statement

Ethical approval for the study was granted by the local ethical committees.

## Funding

The authors have no funding to report.

## Disclosure Statement

Y. Kobari has received financial support from the 10.13039/501100013642Japan Heart Foundation for his fellowship, and speaker fees from Abbott Structural Heart and 10.13039/100008497Boston Scientific. A. Khokhar received honoraria from 10.13039/100000046Abbott, 10.13039/100008497Boston Scientific, and 10.13039/100004374Medtronic. G. Bieliauskas received institutional research grants and consulting fees from 10.13039/100008497Boston Scientific. O. De Backer received institutional research grants and consulting fees from 10.13039/100000046Abbott, 10.13039/100008497Boston Scientific, and 10.13039/100004374Medtronic. The other authors had no conflicts to declare.
