# Supplementary material for: Managing Hostile Aortic Anatomies Using an Extended-Length Introducer Sheath During Transfemoral Transcatheter Aortic Valve Replacement: Rationale and Clinical Outcomes
Source: Struct Heart. 2025 Oct 8;9(12):100737. doi: 10.1016/j.shj.2025.100737 (PMC12664507; doi:10.1016/j.shj.2025.100737)
Supplement: Supplementary Figure 1 [file mmc1.pptx]

## Slide 1
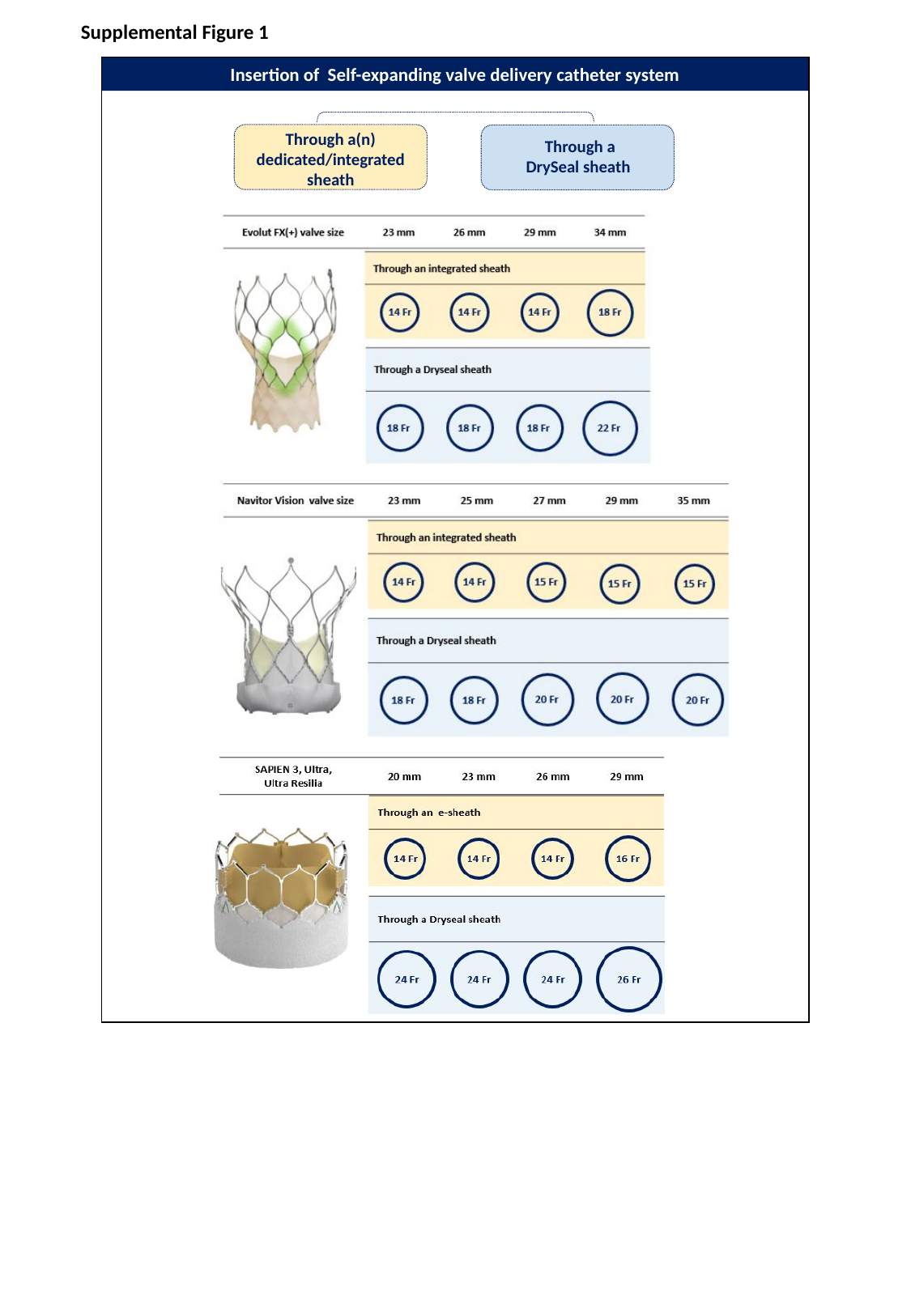

# Supplemental Figure 1
Insertion of Self-expanding valve delivery catheter system
Through a(n)
dedicated/integrated sheath
 Through a
DrySeal sheath
